# Fibrate-based *N*-acylsulphonamides targeting carbonic anhydrases: synthesis, biochemical evaluation, and docking studies

**DOI:** 10.1080/14756366.2019.1611801

**Published:** 2019-05-10

**Authors:** Alessandra Ammazzalorso, Simone Carradori, Andrea Angeli, Atilla Akdemir, Barbara De Filippis, Marialuigia Fantacuzzi, Letizia Giampietro, Cristina Maccallini, Rosa Amoroso, Claudiu T. Supuran

**Affiliations:** a Department of Pharmacy, “G. d’Annunzio” University of Chieti-Pescara, Chieti, Italy;; b Laboratorio di Chimica Bioinorganica, Università degli Studi di Firenze, Florence, Italy;; c Department of Pharmacology, Faculty of Pharmacy, Computer-Aided Drug Discovery Laboratory, Bezmialem Vakif University, Istanbul, Turkey;; d Neurofarba Department, Section of Pharmaceutical and Nutriceutical Sciences, Università degli Studi di Firenze, Florence, Italy

**Keywords:** N-acylsulphonamides, carbonic anhydrase, PPAR antagonist, fibrate derivatives, benzothiazole, benzophenone

## Abstract

A large library of fibrate-based *N*-acylsulphonamides was designed, synthesised, and fully characterised in order to propose them as zinc binders for the inhibition of human carbonic anhydrase (hCA) enzymatic activity. Synthesised compounds were tested against four hCAs (I, II, IX, and XII) revealing a promising submicromolar inhibitory activity characterised by an isozyme selectivity pattern. Structural modifications explored within this scaffold are: presence of an aryl ring on the sulphonamide, *p*-substitution of this aryl ring, benzothiazole or benzophenone as core nuclei, and an *n*-propyl chain or a geminal dimethyl at Cα carbon. Biological results fitted well with molecular modelling analyses, revealing a putative direct interaction with the zinc ion in the active site of hCA I, II and IX. These findings supported the exploration of less investigated secondary sulphonamides as potential hCA inhibitors.

## 1. Introduction

Sulphonamides and their *N*-acyl derivatives represent common functional groups occurring in natural and synthetic drugs. The different biological activities played by these compounds attracted the attention of medicinal chemists, and a great number of lead compounds of pharmaceutical interest were discovered to date^1–3^. Indeed, *N*-acylsulphonamides have been widely used in medicinal chemistry as bioisosteres of carboxylic acids, given the similarity in terms of H-bond properties and acidity^4^. The bioisosteric replacement of carboxylic group with *N*-acylsulphonamide produced novel compounds showing pharmacological activity, sometimes improved with respect to the parent compounds. Paritaprevir (NS3 protease inhibitor)^5^, Venetoclax (Bcl-2 inhibitor)^6^, and Selexipag (prostacyclin receptor agonist)^7^ are *N*-acylsulphonamide drugs recently approved by FDA for the treatment of HCV infection, tumours, and pulmonary arterial hypertension, respectively. A number of *N*-acylsulphonamide derivatives, targeting different enzymes or receptors, have been submitted to clinical or preclinical evaluation: among them, inhibitors of bacterial enzymes^8^^,^^9^, asparagine synthetase inhibitors^10^, agonists and antagonists of nuclear receptor peroxisome proliferator-activated receptors (PPARs)^11^^,^^12^.

Historically, secondary sulphonamides represent an important class of drugs targeting carbonic anhydrases (CAs)^13–16^. These metalloenzymes, catalysing the CO_2_ hydration reaction, are involved in several physiologic functions, also depending on their distribution in different tissues. For these reasons, CA inhibitors present a wide biomedical application, interfering with acid/base regulation, respiration, gluconeogenesis, bone resorption, and tumorigenesis^17^. CA inhibitors, containing the sulphonamide moiety, are therapeutically used as diuretic, antiglaucoma, anticancer, and antiobesity drugs. In recent works, *N*-substituted sulphonamides, among other secondary sulphonamides, have been identified as strong human CA I and II inhibitors, showing nanomolar inhibition constants^18^^,^^19^.

They were characterised by an ionisable moiety as zinc binder directly interacting with the zinc ion in the active site, as demonstrated by X-ray crystallographic studies of human carbonic anhydrase (hCA) II-inhibitor adducts^20^. In the attempt to identify novel CA inhibitors, in this work, we describe the screening of *N*-acylsulphonamide derivatives previously synthesised and identified as PPAR antagonists^21^^,^^22^. These compounds belong to a library of benzothiazole derivatives of fibrates, known PPAR agonists, obtained by an agonist-antagonist switching programme. The inhibitory properties of these derivatives were evaluated on four selected human CA isozymes (I, II, IX, and XII). These isoforms were selected according to their pharmacological relevance for the discovery of innovative therapeutic approaches against hypoxic cancers (hCA IX and XII) and for their wide distribution in the human body (hCA I and II as off-targets). Starting from previously obtained results, a novel series of benzenesulphonamide derivatives were designed, synthesised, and submitted to CA inhibition assays. Furthermore, molecular modelling studies were carried out to explain the putative interactions between this library of heterocyclic compounds and the active sites of the four isozymes in order to rationalise future synthetic approaches within this scaffold and to further understand the structural requirements to obtain isoform selectivity among the tested CAs.

## 2. Experimental protocols

### 2.1. Chemistry

Melting points were determined with a Buchi Melting Point B‐450 and were uncorrected. NMR spectra were recorded on a Varian Mercury spectrometer at 300 MHz or on a Bruker spectrometer at 400 MHz. Proton chemical shifts were referenced to the TMS internal standard. Chemical shifts are reported in parts per million (ppm, δ units). Coupling constants are reported in units of Hertz (Hz). Splitting patterns are designed as s, singlet; d, doublet; t, triplet; q, quartet; dd, double doublet; m, multiplet; and b, broad. All commercial chemicals and solvents were reagent grade and were purchased from Sigma-Aldrich (St. Louis, MO); they are used without further purification, unless otherwise specified. Reactions were monitored by thin layer chromatography on silica gel plates (60 F‐254, Sigma-Aldrich) and the analysis of the plates was carried out using a UV lamp 254/365 nm. Flash chromatography was performed on silica gel 60 (Merck, Kenilworth, NJ). Elemental analyses for C, H, and N were recorded on a Perkin-Elmer 240 B microanalyzer obtaining analytical results within ±0.4% of the theoretical values for all compounds. The following solvents have been abbreviated: chloroform (CHCl_3_), dichloromethane (DCM), diethyl ether (Et_2_O), dimethyl sulphoxide (DMSO), ethanol (EtOH), methanol (MeOH), and tetrahydrofuran (THF).

#### 2.1.1. General procedure for the synthesis of benzenesulphonamides (19–27)

To a stirred solution of fenofibric acid (1.0 mmol) in dry DCM (10 mL) at 0 °C, 1‐ethyl‐3‐[3‐dimethylaminopropyl]carbodiimide hydrochloride (EDC, 1.0 mmol), and 4‐dimethylaminopyridine (DMAP, 1.0 mmol) were added. After 15 min, the selected benzenesulphonamide (1.1 mmol) was added to the reaction mixture and the cold water/ice bath was removed. The reaction mixture was allowed to stir 24 h, diluted with DCM (10 mL), washed with 2N HCl (3 × 20 ml), dried on Na_2_SO_4_, filtered, and evaporated under reduced pressure. Crude products were purified by column chromatography.

##### 2.1.1.1. 2-[4-(4-Chlorobenzoyl)phenoxy]-2-methyl-*N*-[(4-methylphenyl)sulfonyl]propanamide (19)

White solid, 51% yield (silica gel, CHCl_3_/MeOH 98:2); m.p. 185–186 °C; ^1^H NMR (DMSO-d_6_) *δ*1.44 (s, 6H, C(C*H_3_*)_2_), 2.29 (s, 3H, C*H_3_*Ph), 6.66 (d, 2H, *J* 8.7 Hz, C*H*_Ar_), 7.35 (d, 2H, *J* 8.1 Hz, C*H*_Ar_), 7.51 (d, 2H, *J* 8.7 Hz, C*H*_Ar_), 7.60–7.71 (m, 6H, C*H*_Ar_), 12.40 (bs, 1H, N*H*, D_2_O exchange);^13^C NMR (DMSO-*d*_6_) *δ* 21.3, 24.3, 80.9, 117.9, 128.1, 129.1, 129.9, 130.2, 131.6, 132.1, 136.2, 136.5, 137.5, 144.8, 159.0, 172.9, 193.7. Anal. Calcd. for C_24_H_22_ClNO_5_S: C, 61.08; H, 4.70; N, 2.97. Found: C, 60.93; H, 4.69; N, 2.97.

##### 2.1.1.2. 2-[4-(4-Chlorobenzoyl)phenoxy]-*N*-[(4-methoxyphenyl)sulfonyl]-2-methylpropanamide (20)

White solid, 54% yield (silica gel, DCM/MeOH 98:2); m.p. 188–189 °C; ^1^H NMR (CDCl_3_) *δ*1.51 (s, 6H, C(C*H_3_*)_2_), 3.87 (s, 3H, OC*H_3_*), 6.73 (d, 2H, *J* 8.7 Hz, C*H*_Ar_), 6.99 (d, 2H, *J* 8.7 Hz, C*H*_Ar_), 7.46 (d, 2H, *J* 8.7 Hz, C*H*_Ar_), 7.62 (d, 2H, *J* 9.0 Hz, C*H*_Ar_), 7.70 (d, 2H, *J* 9.0 Hz, C*H*_Ar_), 7.93 (d, 2H, *J* 8.7 Hz, C*H*_Ar_), 8.90 (bs, 1H, N*H*, D_2_O exchange); ^13^C NMR (CDCl_3_) *δ* 24.3, 55.7, 81.5, 114.1, 119.0, 128.6, 129.0, 130.9, 131.2, 131.9, 132.1, 135.8, 138.8, 157.4, 164.1, 171.9. Anal. Calcd. for C_24_H_22_ClNO_6_S: C, 59.07; H, 4.54; N, 2.87. Found: C, 59.19; H, 4.53; N, 2.88.

##### 2.1.1.3. 2-[4–(4-Chlorobenzoyl)phenoxy]-*N*-[(4-chlorophenyl)sulfonyl]-2-methylpropanamide (21)

White solid, 69% yield (silica gel, DCM/MeOH 98:2); m.p. 204–205 °C (dec); ^1^H NMR (DMSO-*d*_6_) *δ*1.44 (s, 6H, C(C*H_3_*)_2_), 6.73 (d, 2H, *J* 9.0 Hz, C*H*_Ar_), 7.46–7.69 (m, 9H, C*H*_Ar_, and N*H*, D_2_O exchange), 7.78 (d, 2H, *J* 8.7 Hz, C*H*_Ar_); ^13 ^C NMR (DMSO-*d*_6_) *δ* 24.8, 81.3, 117.8, 128.0, 129.0, 129.3, 129.5, 129.8, 129.9, 131.5, 132.0, 136.7, 137.4, 159.5, 174.0, 193.6. Anal. Calcd. for C_23_H_19_Cl_2_NO_5_S: C, 56.11; H, 3.89; N, 2.84. Found: C, 56.24; H, 3.88; N, 2.84.

##### 2.1.1.4. 2-[4–(4-Chlorobenzoyl)phenoxy]-2-methyl-*N*-[(4-nitrophenyl)sulfonyl]propanamide (22)

White solid, 56% yield (silica gel, DCM/MeOH 98:2); m.p. 196–198 °C; ^1^H NMR (DMSO-*d*_6_) *δ*1.45 (s, 6H, C(C*H_3_*)_2_), 6.71 (d, 2H, *J* 9.0 Hz, C*H*_Ar_), 7.54 (d, 2H, *J* 9.0 Hz, C*H*_Ar_), 7.61 (d, 2H, *J* 8.7 Hz, C*H*_Ar_), 7.68 (d, 2H, *J* 8.7 Hz, C*H*_Ar_), 8.03 (d, 2H, *J* 8.7 Hz, C*H*_Ar_), 8.34 (d, 2H, *J* 8.7 Hz, C*H*_Ar_); ^13^C NMR (DMSO-*d*_6_) *δ*24.8, 81.3, 117.8, 124.5, 129.0, 129.4, 130.0, 131.5, 132.0, 136.6, 137.5, 150.1, 159.4, 174.3, 193.6. Anal. Calcd. for C_23_H_19_ClN_2_O_7_S: C, 54.93; H, 3.81; N, 5.57. Found: C, 55.07; H, 3.79; N, 5.56.

##### 2.1.1.5. *N*-{[4-(acetylamino)phenyl]sulfonyl}-2-[4–(4-chlorobenzoyl)phenoxy]-2-methylpropanamide (23)

White solid, 53% yield (silica gel, DCM/MeOH 98:2); m.p. 226–228 °C; ^1^H NMR (DMSO-*d*_6_) *δ*1.44 (s, 6H, C(C*H_3_*)_2_), 2.03 (s, 3H, C*H_3_*), 6.69 (d, 2H, *J* 8.7 Hz, C*H*_Ar_), 7.52–7.78 (m, 10H, C*H*_Ar_), 10.37 (bs, 1H, N*H*SO_2_, D_2_O exchange), 12.38 (bs, 1H, N*H*Ph, D_2_O exchange); ^13^C NMR (DMSO-*d*_6_) *δ*24.3, 24.5, 80.9, 118.1, 118.6, 129.0, 129.4, 130.4, 131.6, 132.0, 136.5, 137.5, 144.3, 159.0, 169.4, 172.7, 173.4, 193.5. Anal. Calcd. for C_25_H_23_ClN_2_O_6_S: C, 58.31; H, 4.50; N, 5.44. Found: C, 58.44; H, 4.50; N, 5.43.

##### 2.1.1.6. *N*-{4-[({2-[4–(4-chlorobenzoyl)phenoxy]-2-methylpropanoyl}amino)sulfonyl]phenyl} benzamide (24)

White solid, 51% yield (silica gel, CHCl_3_/MeOH 98:2); m.p. 232–234 °C; ^1^H NMR (DMSO-*d*_6_) *δ*1.46 (s, 6H, C(C*H_3_*)_2_), 6.71 (d, 2H, *J* 8.7 Hz, C*H*_Ar_), 7.51–7.68 (m, 9H, C*H*_Ar_), 7.82 (d, 2H, *J* 8.7 Hz, C*H*_Ar_), 7.84–7.98 (m, 4H, C*H*_Ar_), 10.64 (bs, 1H, N*H*SO_2_, D_2_O exchange), 12.45 (bs, 1H, N*H*Ph, D_2_O exchange); ^13^C NMR (DMSO-*d*_6_) *δ*24.4, 81.0, 118.2, 120.1, 126.9, 128.3, 128.9, 129.3, 130.5, 131.6, 132.4, 134.7, 136.5, 137.6, 139.2, 142.6, 144.4, 159.0, 166.5, 172.8, 193.6. Anal. Calcd. for C_30_H_25_ClN_2_O_6_S: C, 62.44; H, 4.37; N, 4.85. Found: C, 62.35; H, 4.36; N, 4.86.

##### 2.1.1.7. 2-[4-(4-Chlorobenzoyl)phenoxy]-2-methyl-*N*-({4-[(phenylacetyl)amino]phenyl}sulphonyl) propanamide (25)

White solid, 47% yield (silica gel, DCM/MeOH 98:2); m.p. 170–172 °C; ^1^H NMR (DMSO-*d*_6_) *δ*1.43 (s, 6H, C(C*H_3_*)_2_), 3.64 (s, 2H, C*H_2_*), 6.72 (d, 2H, *J* 8.1 Hz, C*H*_Ar_), 7.19–7.30 (m, 5H, C*H*_Ar_), 7.55–7.76 (m, 10H, C*H*_Ar_), 10.63 (bs, 1H, N*H*SO_2_, D_2_O exchange); ^13^C NMR (DMSO-*d*_6_) *δ*24.4, 43.7, 81.0, 118.3, 118.9, 127.1, 128.8, 129.1, 129.5, 129.6, 131.6, 132.0, 135.8, 136.5, 144.2, 159.0, 170.3, 172.8, 193.6. Anal. Calcd. for C_31_H_27_ClN_2_O_6_S: C, 62.99; H, 4.60; N, 4.74. Found: C, 62.89; H, 4.61; N, 4.75.

##### 2.1.1.8. 2-(4-Benzoylphenoxy)-*N*-(phenylsulphonyl)acetamide (26)

White solid, 59% yield (silica gel, DCM); m.p. 61–62 °C; ^1^H NMR (CDCl_3_) *δ*4.55 (s, 2H, C*H_2_*), 6.95 (d, 2H, *J* 8.7 Hz, C*H*_Ar_), 7.45–7.83 (m, 5H, C*H*_Ar_), 7.66–7.83 (m, 5H, C*H*_Ar_), 8.10 (d, 2H, *J* 7.5 Hz, C*H*_Ar_), 9.06 (bs, 1H, N*H*, D_2_O exchange); ^13^C NMR (CDCl_3_) *δ*77.2, 114.4, 126.4, 128.3, 129.0, 129.1, 129.8, 132.3, 132.6, 132.8, 137.6, 142.0, 159.7, 195.2. Anal. Calcd. for C_21_H_17_NO_5_S: C, 63.79; H, 4.33; N, 3.54. Found: C, 64.02; H, 4.32; N, 3.53.

##### 2.1.1.9. 2-(4-Benzoylphenoxy)-*N*-(phenylsulphonyl)pentanamide (27)

White solid, 49% yield (silica gel, DCM); m.p. 140–142 °C; ^1^H NMR (CDCl_3_) *δ*0.87 (t, 3H, *J* 6.9 Hz, C*H_3_*), 1.36–1.43 (m, 2H, C*H_2_*), 1.82–1.91 (m, 2H, C*H_2_*), 4.58 (t, 1H, *J* 6.0 Hz, C*H*), 6.83 (d, 2H, *J* 9.0 Hz, C*H*_Ar_), 7.46–7.65 (m, 6H, C*H*_Ar_), 7.71–7.76 (m, 4H, C*H*_Ar_), 7.95 (d, 2H, *J* 7.2 Hz, C*H*_Ar_), 8.81 (bs, 1H, N*H*, D_2_O exchange); ^13 ^C NMR (CDCl_3_) *δ*13.5, 17.8, 34.1, 78.6, 114.7, 126.4, 128.3, 129.0, 129.8, 132.0, 132.3, 132.6, 134.2, 137.6, 137.9, 159.6, 169.3, 195.2. Anal. Calcd. for C_24_H_23_NO_5_S: C, 65.89; H, 5.30; N, 3.20. Found: C, 65.91; H, 5.29; N, 3.21.

#### 2.1.2. General procedure for the synthesis of esters (29 and 31)

A solution of sodium (2.5 mmol) in absolute EtOH (10 ml) was added to 4-hydroxybenzophenone (2.5 mmol), dissolved in absolute EtOH (10 ml), at room temperature, and under nitrogen atmosphere. Ethyl 2-bromoacetate (or ethyl 2-bromovalerate) (2.5 mmol) in absolute EtOH (5 ml), was added to the mixture, and the resulting solution was refluxed for 20 h. After evaporation of solvent under reduced pressure, the residue was poured into water (20 ml) and extracted with Et_2_O (3 × 20 mL). The organic layer was dried over Na_2_SO_4_ and concentrated under reduced pressure.

##### 2.1.2.1. Ethyl (4-benzoylphenoxy)acetate (29)

White solid, 63% yield. Characterisation data were in agreement with those reported in the literature^23^.

##### 2.1.2.2. Ethyl 2–(4-benzoylphenoxy)pentanoate (31)

Pale yellow oil, 69% yield (silica gel, DCM); ^1^H NMR (CDCl_3_) *δ*0.98 (t, 3H, *J* 6.9 Hz, C*H_3_*), 1.25 (t, 3H, *J* 6.9 Hz, C*H_3_*), 1.49–1.63 (m, 2H, C*H_2_*), 1.83–2.08 (m, 2H, C*H_2_*), 4.23 (q, 2H, *J* 7.2 Hz, OC*H_2_*), 4.70 (dd, 1H, *J* 7.8 Hz, *J* 7.5 Hz, C*H*), 6.92 (d, 2H, *J* 8.7 Hz, C*H*_Ar_), 7.43–7.48 (m, 2H, C*H*_Ar_), 7.52–7.59 (m, 1H, C*H*_Ar_), 7.75 (d, 2H, *J* 7.2 Hz, C*H*_Ar_), 7.79 (d, 2H, *J* 7.2 Hz, C*H*_Ar_); ^13^C NMR (CDCl_3_) *δ* 13.6, 14.1, 18.5, 34.6, 61.4, 76.2, 114.4, 128.2, 129.7, 130.7, 131.9, 132.5, 138.0, 161.4, 171.2, 195.5. Anal. Calcd. for C_20_H_22_O_4_: C, 73.60; H, 6.79; Found: C, 73.52; H, 6.81.

#### 2.1.3. General procedure for the synthesis of acids (30 and 32)

2N NaOH (12.5 mmol) was added to esters **29** and **31** (1.5 mmol) in THF (10 ml) and the mixture was stirred at r.t. overnight. THF was removed under reduced pressure; the solution was acidified by 4N HCl, obtaining a precipitate that was collected by filtration under vacuum.

##### 2.1.3.1. (4-Benzoylphenoxy)acetic acid (30)

White solid, 72% yield. Characterisation data were in agreement with those reported in the literature^23^.

##### 2.1.3.2. 2-(4-Benzoylphenoxy)pentanoic acid (32)

White solid, 99% yield; m.p. 75–77 °C; ^1^H NMR (CDCl_3_) *δ*0.98 (t, 3H, *J* 7.2 Hz, C*H_3_*), 1.50–1.63 (m, 2H, C*H_2_*), 1.96–2.05 (m, 2H, C*H_2_*), 4.75 (dd, 1H, *J* 7.8 Hz, *J* 7.8 Hz, C*H*), 6.93 (d, 2H, *J* 8.7 Hz, C*H*_Ar_), 7.43–7.48 (m, 2H, C*H*_Ar_), 7.53–7.59 (m, 1H, C*H*_Ar_), 7.74 (d, 2H, *J* 7.2 Hz, C*H*_Ar_), 7.81 (d, 2H, *J* 7.2 Hz, C*H*_Ar_); ^13^C NMR (CDCl_3_) *δ* 13.6, 18.5, 34.5, 75.6, 114.4, 128.2, 129.8, 130.9, 132.1, 132.6, 137.9, 161.2, 175.9, 195.7. Anal. Calcd. for C_18_H_18_O_4_: C, 72.47; H, 6.08; Found: C, 72.51; H, 6.07.

### 2.2. CA inhibition assays

An Applied Photophysics stopped-flow instrument has been used for assaying the CA catalysed CO_2_ hydration activity. Phenol red (0.2 mM) has been used as indicator, working at the absorbance maximum of 557 nm, with 20 mM Hepes (pH 7.5, for α-CAs) as buffer, and 20 mM NaClO_4_ (for maintaining constant the ionic strength), following the initial rates of the CA-catalysed CO_2_ hydration reaction for a period of 10–100 s. The CO_2_ concentrations ranged from 1.7 to 17 mM for the determination of the kinetic parameters and inhibition constants. In particular, CO_2_ was bubbled in distilled deionised water for 30 min till saturation. A CO_2_ kit (Sigma, Milan, Italy) was used to measure the concentration in serially diluted solutions from the saturated one at the same temperature. For each inhibitor at least six traces of the initial 5–10% of the reaction have been used for determining the initial velocity. The uncatalysed rates were determined in the same manner and subtracted from the total observed rates. Stock solutions of inhibitor (1 µM) were prepared in distilled–deionised water and dilutions up to 0.1 nM were done thereafter with the assay buffer. Inhibitor and enzyme solutions were preincubated together for 15 min at room temperature prior to assay, in order to allow for the formation of the E–I complex or for the eventual active site mediated hydrolysis of the inhibitor. The inhibition constants were obtained by non-linear least-squares methods using PRISM 3 and the Cheng–Prusoff equation and represent the average from at least three different determinations. All recombinant CA isoforms were obtained in-house as previously reported^24^^,^^25^.

### 2.3. Molecular modelling studies

Crystal structures of hCA I (pdb: 3lxe, 1.9 Å, in complex with topiramate), hCA II (pdb: 4e3d, 1.6 Å, in complex with 2,5-dihydroxybenzoic acid), hCA IX (pdb: 3iai, 2.2 Å, in complex with acetazolamide [AAZ]) and XII (pdb: 1jd0, 1.5 Å, in complex with AAZ) were obtained from the Brookhaven Protein Data Bank. All ligands (AAZ, topiramate and 2,5-dihydroxybenzoic acid) and the zinc-bound water molecule of the hCA II structure were retained and all other non-protein atoms were deleted. Chain A was retained if more than one protein structure was present in the crystal structure. Hydrogen atoms were added with the “protonate 3 D” tool and subsequently a steepest-descent energy minimisation was performed using the AMBER14:EHT force field (MOE software package, version 2018.0101, chemical computing group, Inc., Montreal, Canada)^26^. The four protein structures were superposed on the backbone atoms of hCA I (Cα atoms, RMSD: 1.395 Å, for 236 residues). The coordinates of the hCA II zinc-bound water molecule were copied into the other hCA structures.

The molecular structures of the ligands were prepared with the MOE software package. All stereoisomers were generated. Subsequently, the ligand structures were energy minimised (MMFF94x force field) and the ligands were saved as multi-mol2 files.

Docking calculations were performed with the GOLD software package version 5.6.1 (CCDC, Cambridge, UK) using the ChemScore scoring function (25 genetic algorithm runs per ligand) and default settings. The binding pocket was defined as within 14 Å around a centroid (x: −18.899; Y: 36.167; z: 45.640; corresponds to the AAZ O_3_ atom of the hCA IX structure after superposition upon the hCA I structure). Dockings into the active site were performed either with or without a zinc-bound water molecule (obtained from hCA II structure 4e3d)^27^.

## 3. Results and discussion

### 3.1. Synthetic approach and *in vitro* CA inhibition studies

A wide group of benzothiazole compounds, bearing an *N*-acylsulphonamide portion, have been firstly synthesised by our research group and tested as PPAR antagonists. Some of them were also studied for assessing antitumor effects, mainly in cancer models overexpressing PPARα^28^^,^^29^. We decided to evaluate their inhibitory effect on four selected human CA isoenzymes (I, II, IX, and XII) and AAZ was used as reference compound. A preliminary screening of compounds **1–7**, methane and benzenesulphonamide derivatives, was carried out, and the results are reported in Table 1. The greater affinities showed by benzenesulphonamide derivatives prompted us to further explore benzenesulphonamide derivatives such as *p*-substitutions on the aromatic ring (**8–14**). In addition, we also tested compounds **15–18**, previously synthesised starting from the parent compounds clofibrate, gemfibrozil, bezafibrate, and fenofibrate^30^.

**Table 1.** Inhibitory activity of derivatives **1–18** and reference compound acetazolamide (**AAZ**) against four selected hCA isoforms (I, II, IX, and XII) by stopped-flow CO_2_ hydrase assay^31–35^.

The three methanesulphonamide derivatives (compounds **2**, **4**, **6**) showed no activity against CA except for compound **2** which had a slight activity against hCA XII (*K*_i _= 61.0 μM), whereas corresponding benzenesulphonamides (**1**, **3**, **5**) moderately inhibited CA I, II, and IX, with a slight preference for CA II. Compounds **1–4** and **6–7**, bearing an alkylic chain on Cα (adjacent to carbonyl group), produced an improved CA inhibition compared to phenyl derivative **5**.

The series of compounds **8**–**14** includes *p*-substituted benzenesulphonamides, obtained starting from compound **7**. The best inhibition profiles against CA were found for *p*-methoxy derivative **9** and *p*-chloro derivative **10**. They produced a selective inhibition with submicromolar *K*_i_ values against hCA IX and hCA II respectively. The insertion of methyl (**8**), nitro (**11**), acetylamino (**12**), benzoylamino (**13**) and phenylacetylamino (**14**) groups did not improve their inhibition properties.

Benzenesulphonamides **15**–**18**, obtained by their carboxylic precursors, were all inactive against CA XII. Compounds **15** and **16** (clofibrate and gemfibrozil derivatives) were completely inactive against all tested isoenzymes, whereas **17** and **18** showed a moderate inhibition against CA I, II and IX. The best inhibition values were found for **18** (fenofibrate derivative), that showed submicromolar *K*_i_ inhibition against I and IX isoenzymes.

**Figure 1.**
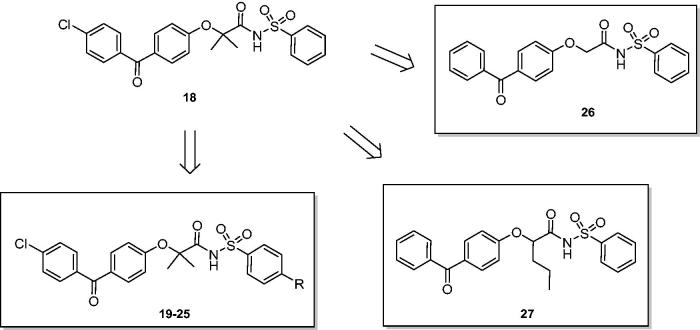
From lead compound **18** to novel benzenesulphonimides **19–27**.

**Scheme 1.**
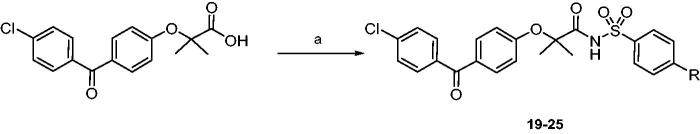
Reagents and conditions: (a) *p*-substituted benzenesulphonamide, EDC, DMAP, dry dichloromethane, 0 °C-r.t., 24 h.

According to the above reported results, we selected fenofibrate derivative **18** as a novel lead compound to develop novel chemical derivatives, by adding substituents in *para* position to the benzenesulphonamide moiety (Figure 1) such as methyl, methoxy, chlorine, nitro, acetylamino, benzoylamino, and phenylacetylamino groups. Compounds **19–25** were easily synthesised by reacting fenofibric acid, obtained from hydrolysis of fenofibrate, with *p*-substituted benzenesulphonamides, in the presence of 1-ethyl-3–(3-dimethylaminopropyl)carbodiimide (EDC) and 4-dimethylaminopyridine (DMAP), in dry dichloromethane and under nitrogen atmosphere (Scheme 1).

With the aim to evaluate the effect of steric hindrance on Cα, we planned the synthesis of **26** and **27** (Figure 1), where the Cα was unsubstituted or substituted with an *n*-propylic chain; both derivatives are lacking of chlorine substitution on benzophenone ring.

**Scheme 2.**
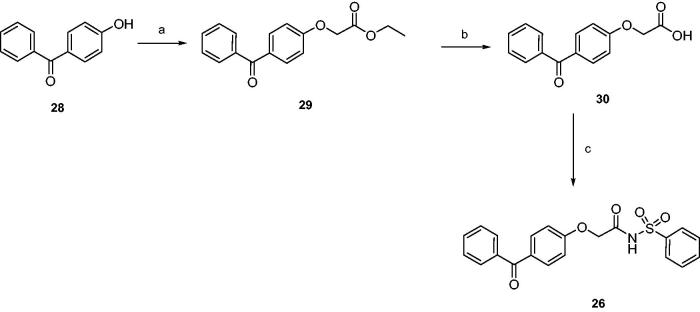
Reagents and conditions: (a) ethyl 2-bromoacetate, sodium, absolute ethanol, reflux, 20 h; (b) 2N NaOH, THF, r.t., 24 h; (c) benzenesulphonamide, EDC, DMAP, dry dichloromethane, 0 °C-r.t., 24 h.

**Scheme 3.**
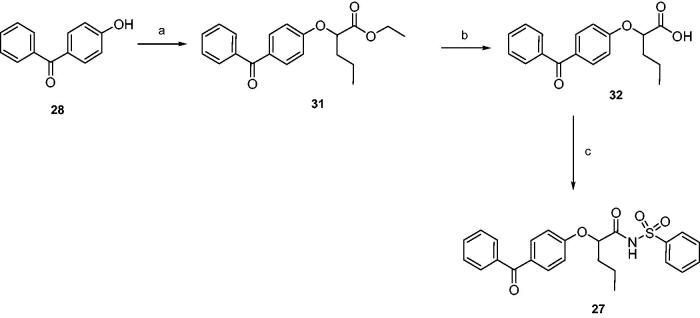
Reagents and conditions: (a) ethyl 2-bromovalerate, sodium, absolute ethanol, reflux, 20 h; (b) 2N NaOH, THF, r.t., 24 h; (c) benzenesulphonamide, EDC, DMAP, dry dichloromethane, 0 °C-r.t., 24 h.

Finally, products **26** and **27** were synthesised as depicted in Schemes 2–3. Esters **29** and **31** were obtained by nucleophilic displacement of proper ethyl 2-bromoalkanoates by 4-hydroxybenzophenone; hydrolysis by 2N NaOH afforded acids **30** and **32**, that were coupled with benzenesulphonamide as previously described.

Compounds **19–27** were also screened against CA I, II, IX, and XII to derive robust SAR within this scaffold: results are shown in Table 2.

**Table 2.** Inhibitory activity of derivatives **19–27** and reference compound acetazolamide (AAZ) against four selected hCA isoforms (hCA I, II, IX, and XII) by stopped-flow CO_2_ hydrase assay^31^^,^^32^.

Overall, the *p*-substitution of aromatic ring with respect to parent derivative **18** did not improve hCA inhibition, except for compounds **22** and **24**, showing both a marked inhibition of hCA II (*K*_i _=16 and 77 nM, respectively), and a good selectivity *versus* this isoform. The presence of an acetylamino group was detrimental for hCA inhibition (**23**), whereas the introduction of an additional aromatic ring (**24**) produced a marked effect on both hCA I and hCA II. The further elongation of the substituent (**25**) decreased the inhibitory effect. Compounds **26** and **27**, lacking of the chlorine atom on benzophenone structure and showing a different steric hindrance on Cα, did not induce a sensible inhibition of tested isoenzymes.

### 3.2. Molecular modelling studies

#### 3.2.1. Docking studies into the active site of hCA I

**Figure 2.**
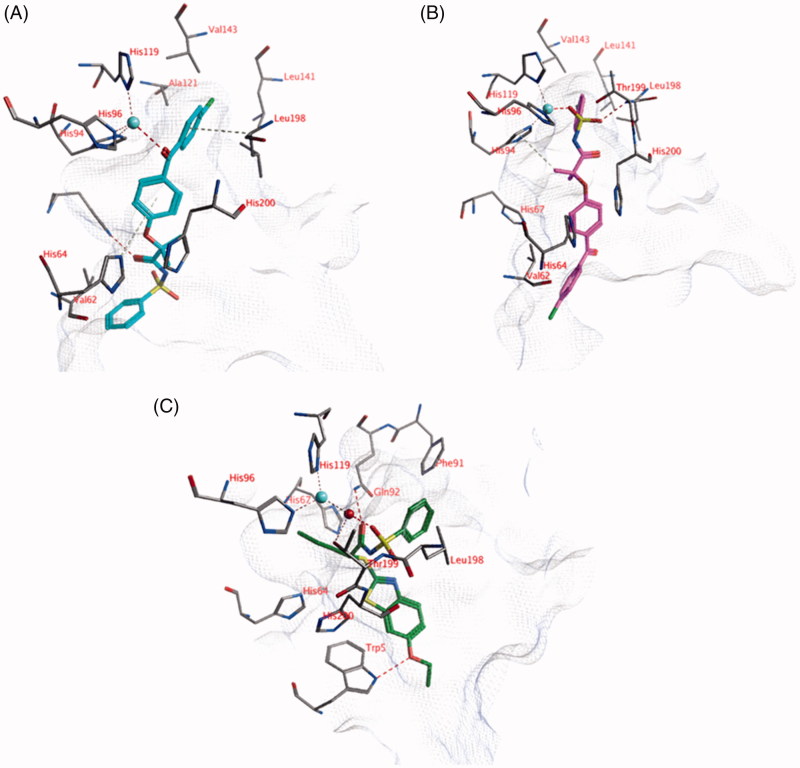
(A) The docked pose of compound **18** (turquoise), (B) compound **21** (purple) and (C) compound **1** (*R*-isomer; green) in the active site of hCA I (pdb: 3lxe). Hydrogen bonds and interactions to the active site zinc ion are indicated in red dashed lines. Aromatic system – H bonds are indicated in yellow dashed lines.

The ligands were docked into the active site of hCA I with and without a zinc-bound water molecule. This water molecule was obtained from the hCA II structure (pdb: 4e3d) as described in the Materials and Methods section. Compounds **3**, **18**, **21,** and **24** show lower *K*_i_ values than 10 μM (Tables 1 and 2). Two different poses have been identified in which the ligands can directly interact with the active site zinc ion. In the first pose, the carbonyl group located between the two phenyl moieties forms an interaction with the active site zinc ion (Figure 2(A)). The two phenyl groups form hydrophobic interactions with His64, Ala121, Val143, Leu198, His200, and Val207. A second hydrogen bond is formed between the other carbonyl atom of the ligand and the side chain of His67. The terminal unsubstituted phenyl group forms a hydrophobic interaction with the side chain of Val62. In addition, hydrogen atoms of His64 and Leu198 point towards the centroids of the phenyl rings resulting in aromatic-H bonds. The sulphonamide group is solvent exposed. This pose is observed for many compounds, including compounds **18** and **24**.

In the second docked pose, the sulphonamide moiety is located close the active site zinc ion (Figure 2(B)). One of the sulphonamide oxygen atoms forms an interaction with the zinc ion, while the other oxygen atom forms a hydrogen bond with the backbone of Thr199. The chlorine-substituted phenyl group forms hydrophobic interactions with Ala121, Leu141, Val143, and Leu198. One of the ligand’s hydrogen atoms points towards the centre of His94 to form aromatic-H bonds. No additional hydrogen bonds are observed between the ligand and the enzyme, however, the amide bond and the carbonyl group of the ligand are solvent accessible and may form interaction with water. This pose is observed for many compounds, including compounds **3**, **18,** and **21**.

Docked poses have been identified in which the ligands directly interact with the zinc-bound water molecule. For example, the *R* isomer of compound **1** forms a hydrogen bond with the water molecule through its sulphonamide oxygen atom (Figure 2(C)). The carbonyl group of the ligand forms a hydrogen bond with the side chain of Gln92. A third hydrogen bond is formed between the ligand and the side chain of Trp5. The propyl group of the ligand is located in a hydrophobic pocket formed by Val62, His64, His67, and His200. The phenyl group of the ligand forms hydrophobic interactions with Phe91, Leu131, Leu141, and Leu198. Interestingly, similar docked poses have not been obtained for the *S* isomer of compound **1**. Similar observations have been made for the other compounds in which some isoforms may bind to one stereoisomer but not to the other.

#### 3.2.2. Docking studies into the active site of hCA II

**Figure 3.**
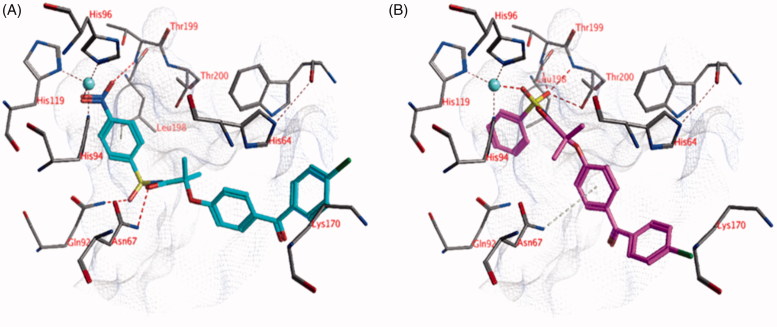
The docked pose of (A) compound **22** (turquoise) and (B) compound **18** (purple) in the active site of hCA II (pdb: 4e3d). Hydrogen bonds and interactions to the active site zinc ion are indicated in red dashed lines. Aromatic system – H bonds are indicated in yellow dashed lines.

The docked pose of compound **22** (*K*_i _=16 nM) suggests that the nitro group could be located near the zinc ion (Figure 3(A)). The partially negatively charged oxygen atoms of the nitro group may form a hydrogen bond with the backbone of Thr199 and electrostatic interactions with the zinc ion. One of the sulphonamide oxygen atoms forms a hydrogen bond with the side chain of Gln92, while the other oxygen atom is water-accessible. The ligand amide group forms a hydrogen bond with the side chain of Asn67 and the chlorine substituent is close enough to the side chain of Lys170 for electrostatic interactions (distance = 4.5 Å). Hydrophobic interactions are formed mainly with Leu198. All hydrogen bond donors and acceptors of the ligand that do not interact with the active site are water-accessible.

The docked pose of compound **18** (*K*_i _= 1.3 μM) indicates that the sulphonamide moiety of the ligand is located near the zinc ion (Figure 3(B)). One of the sulphonamide oxygen forms a direct interaction with the zinc ion, while the other oxygen atom may form hydrogen bonds with Leu198 (backbone) and/or Thr200 (backbone and side chain). One of the side chain hydrogen atoms of Asn67 points towards the centroid of the ligand phenyl group, which could lead to aromatic-H interactions. The chlorine substituent may form electrostatic interactions with Lys170 (distance =5.4 Å).

Many docked poses of the other ligands show a similar position for the sulphonamide group as obtained for compound **18**.

#### 3.2.3. Docking studies into the active site of hCA IX

**Figure 4.**
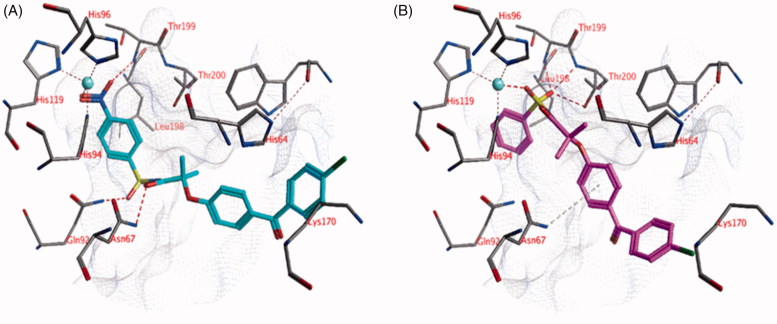
The docked pose of (A) compound **18** (purple) and (B) alternative docked pose of compound **18** (purple) in the active site of hCA IX (pdb: 3iai). Hydrogen bonds and interactions to the active site zinc ion are indicated in red dashed lines. Aromatic system – H bonds are indicated as yellow dashed lines.

The docked pose for compound **18** (*K*_i_ = 0.44 μM) indicates that the sulphonamide may be located close to the zinc ion, enabling one oxygen to form an interaction with the zinc ion (tetrahedral orientation around zinc) and the other oxygen atom to form hydrogen bonds with Thr200 (Figure 4(A)). The carbonyl group adjacent to the sulphonamide moiety is located close to the zinc ion and interaction may occur, resulting in a distorted trigonal bipyramidal orientation around zinc. The terminal phenyl moiety forms hydrophobic interactions with Val121, Val143, and Leu198. The central phenyl group forms hydrophobic interactions with Trp5, while the substituted phenyl group forms hydrophobic interactions with Pro202. A similar docked pose as described for compound **18** or other docked poses in which the ligand directly interacts with the zinc ion are not observed for most of the compounds.

An alternative docked pose has been obtained for compound **18** in which it interacts with the zinc-bound water molecule *via* one of its sulphonamide oxygen atoms (Figure 4(B)). The other oxygen atom forms a hydrogen bond to the side chain of Gln92. A second hydrogen bond is formed with the side chain of Trp5. The ligand phenyl group forms hydrophobic interactions with Pro202, while the two methyl groups are located in a hydrophobic cavity formed by Val131, Leu135, Leu141, and Leu198.

#### 3.2.4. Docking studies into the active site of hCA XII

No docking poses have been obtained in which the ligands interact directly with the zinc ion or the zinc-bound water molecule.

## 4. Conclusions

This library of fibrate-based *N*-acylsulphonamides supported the exploration of other zinc binders for the inhibition of human CAs with respect to the widely studied primary sulphonamides. These secondary sulphonamides maintained the possibility to interact with the zinc ion through several binding modes. As also reported in previous crystallographic studies, they present a reduced submicromolar inhibitory activity, but they gained an interesting isoform selectivity against the four tested hCAs. With the support of molecular modelling studies, we assessed the structural requirements within this scaffold to further improve the biological activity such as the presence of an aryl ring on the sulphonamide, *p*-substitution of this aryl ring, benzothiazole or benzophenone as tailing moieties, and an *n*-propyl chain or a geminal dimethyl at Cα carbon.
